# Supplementary material for: Imaging the dynamics of cardiac fiber orientation in vivo using 3D Ultrasound Backscatter Tensor Imaging
Source: Sci Rep. 2017 Apr 11;7:830. doi: 10.1038/s41598-017-00946-7 (PMC5429761; doi:10.1038/s41598-017-00946-7)
Supplement: Supplementary file 1 — supplementary video caption [file 41598_2017_946_MOESM1_ESM.doc]

**Imaging the dynamics of cardiac fiber orientation in vivo using 3D Ultrasound Backscatter Tensor Imaging**

Clement Papadacci^1§^, Victor Finel^1§^, Jean Provost^1^, Olivier Villemain^1^, Patrick Bruneval^2^, Jean-Luc Gennisson^1^, Mickael Tanter^1^, Mathias Fink^1^, Mathieu Pernot^1*^

Supplementary video caption

**In vivo 3D-BTI movie of the left ventricular wall in a beating heart.** 3D representation of fibers orientation and its dynamics in the left ventricular wall of an open-chest sheep during one cardiac cycle.
